# “My Friends are at the Bottom of My Schedule”: A Qualitative Study on Social Health among Nursing Students during Clinical Placement

**DOI:** 10.3390/ijerph17186921

**Published:** 2020-09-22

**Authors:** Hon Lon Tam, Aimei Mao, Pak Leng Cheong, Iat Kio Van

**Affiliations:** Education department, Kiang Wu Nursing College of Macau, Macau 999078, China; maoaimei@kwnc.edu.mo (A.M.); joecheong@kwnc.edu.mo (P.L.C.); van@kwnc.edu.mo (I.K.V.)

**Keywords:** qualitative study, social health, nursing student, clinical placement

## Abstract

Clinical placement is an essential component for nursing students, allowing them to transfer professional knowledge into practice. The quality of life among nursing students and nurses was reviewed to examine its impact on the quality of provided care. However, it is unclear how social health among nursing students is affected during clinical placement. Final-year students who had finished clinical placement were invited to participate in this qualitative study. Twenty-one in-depth interviews were conducted and transcribed verbatim for thematic analysis. Two main themes, i.e., contributors to lack of social health, and manifestations of lack of social health, emerged from seven sub-themes. Students experienced different challenges during the clinical placement, but some of these did contribute to effects on their social health. Lack of social health might further influence career development after graduation. Supportive strategies from colleagues, nursing colleges and hospitals might potentially improve students’ social health during the clinical placement.

## 1. Introduction

Clinical placement is an important experience for nursing students to transfer professional knowledge from theory into practice [1] and to learn how to act as a nurse in future [2,3]. Students reflect on the care they provide during clinical placement to facilitate the learning process [4]. Different specialties such as medical ward, surgical ward, emergency room and pediatric ward ae covered during clinical placements to allow the students to learn from different experiences. Different duration of clinical placement for different specialties are also required for nurse registration. In Macau, a minimum of 1840 h of clinical placement is arranged in a pre-registered nursing program. The total of 1840 h is split into the whole length of a four-year baccalaureate program, with 80 h in the first year, 320 h in the second year, 480 h in the third year and 960 h in the final year. During the clinical placement, nursing students develop their professional identity, and the values of nursing are transferred implicitly by their clinical preceptors [5,6]. Numerous studies have explored the process and characteristics of professional identity construction of nursing students and nurses [7]. Contrary to the well-researched area on professional identity construction, research is scarce on the specific aspects of the nursing students’ daily life impacted upon by the identity construction process. The construction of their professional identity is challenging for students in many circumstances and may adversely affect the student’s quality of life (QOL).

## 2. Literature Review

### 2.1. QOL among Nursing Students and Nurses

The World Health Organization (WHO) defines QOL by looking at individuals’ perception of their position in life. The QOL assessment tool developed by the WHO is composed of four domains: physical, psychological, social and environmental [8]. The physical health domain assesses satisfaction in daily activities such as sleep, energy level and dependence on medical substances. Individual feelings, appraisal of bodily image and appearance are assessed in the psychological health domain. The social health domain contains personal relationships with family and friends, social support from surrounding people, and sexual activities. The environmental health domain assesses the satisfaction of surrounding factors such as financial resources, safety, accessibility and quality of health and social care. The WHO highlights that QOL is constructed within the different contexts and value systems in which individuals live [8].

During clinical placement, fatigue and psychological symptoms, such as distress, anxiety, and depression are frequently reported by medical and nursing students, and these challenges may influence their QOL [9,10]. These students often experience a difficult transition time as, in many cases, they have to manage patient care independently in a complicated clinical environment [3,11,12,13]. When comparing nursing students in different years, the final-year nursing students in clinical placement had worsened QOL in the domains of physical and social health [14]. A range of factors were identified as influencing QOL among nursing students and nurses, including personal features and socioeconomic context. Cruz et al. employed the WHO developed QOL assessment tool to assess QOL among nursing students in nine countries [9]. A total of 2012 students were recruited and the results indicated that age, residence and family income contributed to the QOL of nursing students. Nowrouzi et al. reported other contributory influences on nurse’s QOL including personal lifestyle systems and procedures, the health care system, the workplace system and the financial compensation system, with each of the four systems containing a variety of factors [15]. For instance, the workplace system includes job position, department, organization and external environment. The authors called for changes within the workplace and across the health care system to improve nurses’ QOL [15]. Given that QOL is multi-dimensional, the factors influencing it are multi-faceted. Nurses and nursing students were found to apply various strategies to cope with the difficulties they encountered in order to improve their QOL, such as self-adaptation and pursuit of social support. As their social environment is an integral component of QOL, nursing students and nurses seek social support to improve their QOL.

### 2.2. Social Health Among Nursing Students and Nurses

Social health refers to an individual’s ability to foster satisfying interpersonal relationships with others. While relationships with others and social support are highlighted in the domain of social health by the WHO, Keyes suggested that the status of an individual’s social health relates to five challenges that an individual encounters when he/she engages in society, i.e., social integration, social acceptance, social contribution, social actualization and social coherence [16]. Social integration is an individual’s sense of belonging to a group; social acceptance is the individual’s feeling of being accepted by the group members and his/her attitudes towards the group; social contribution is the sense of one’s usefulness to the group; social actualization refers to the individual’s perception of the future development of the group, showing his/her confidence in the group; social coherence refers to the individual’s assessment of the quality and functioning of the group [16]. Mozaffari and colleagues interviewed 18 nurses with various clinical experiences in Iran and advocated that holistic support was needed to improve social health among nurses, which included support from family, colleagues, and the organization as well as from society and the media [17].

Only limited studies have reported on social health among nursing students. Cruz et al. revealed that nursing students had the lowest score in the domain of social health compared to other domains [9]. However, another study using the same QOL assessment tool on 444 baccalaureate nursing students in the Philippines reported that social health was rated as the highest score [18]. The Filipino study attributed the high level of social health to the right social support enjoyed by the nursing students; whereas Cruz et al. blamed the low level of social health on the busy academic life endured by the nursing students [9]. Thus, a supportive system in academic life may help nursing students to address the issues of social health.

Health is a state of physiological, psychological and social wellbeing [19]. These components interact to contribute to overall health status. For instance, if the individual is not satisfied by encounters with other people, he/she will feel isolated resulting in poor social health. A low level of social health can influence psychological wellbeing and cause physical symptoms eventually [20,21]. However, few studies have specifically focused on social health and its contributors.

In brief, different physical and psychological symptoms can be developed during clinical placement. Previous studies have explored QOL in the dimensions of physical and psychological health, but few have specifically explored the component of social health. Macau is a small city but is ranked in first place in the world for high density of population and sixth place for gross domestic product per capita [22,23]. This qualitative research study will explore nursing students’ social health and contributing factors. By targeting final-year nursing students in a specific socioeconomic context, this study will contribute knowledge relating to the characteristics of social health at a critical time of professional development.

## 3. Materials and Methods

The study was a qualitative study using in-depth interviews. A qualitative approach was chosen as this could provide further understanding of students’ unique experience on the clinical placement. An interview guide was developed from the literature and from experts in nursing education in Macau to explore what impressed about the placement, what difficulties were suffered during the placement and how were these managed (see interview guide in Supplementary Materials). The study was reported in accordance with the Consolidated Criteria for Reporting Qualitative research (COREQ) guidelines [24].

### 3.1. Method and Participants

All interviews were conducted by the same interviewer between April and June 2017. The interviewer was a lecturer in the nursing college who was not involved in any teaching activity of the final-year students and had no conflict with their placement results. At the beginning, three interviews were supervised by a senior qualitative researcher to ensure they met the aim of the study. The senior qualitative researcher was a nurse educator having more than twenty years’ experience in teaching and clinical practice. Field notes were made in each interview, which included the time and the place of the interview conducted, interactions between interviewer and interviewee, and the feelings of the interviewer towards the interview. Data saturation was suggested if no new information emerged from three consecutive interviews.

All participants were from a four-year undergraduate nursing program in a nursing college in Macau. Final-year students were chosen because half of the clinical placement in the program, 960 h, was arranged in the fourth year, with the result that students would already have a strong impression of the placement experience. The students were invited immediately after they had finished clinical placement at the end of March 2017. The time of recruitment was carefully considered in order to focus on students’ clinical placement experience and minimize interruptions to their study. Convenience sampling was used and invitations were made by the research team during a post-placement student gathering. Ten students expressed willingness to participate, and the remaining participants were invited by telephone. Calls were made in ascending order of student number. Each student was called once whether he/she answered or not. Once the participant had answered the call, the aim of the study was explained briefly and the interview was scheduled according to the participant’s availability once he/she had agreed to participate in the study.

#### Participant Sample Characteristics

The characteristics of the participants are shown in Table 1. All participants were female since there was only one male student in their class. There were 59 students in the class, and 14 refused to participate in the study because of lack of interest or unavailability. The rest were not called, as data saturation had been reached. Few participants were nominated by secondary schools, and less than half of the participants ranked nursing as their first choice of study in tertiary education.

### 3.2. Ethical Considerations

Ethical approval was obtained from the college research committee with reference number 2016JAN01. A written participant information sheet was given to each participant prior to consent with the emphasis on voluntary participation, confidentiality and anonymity. Ample time for consideration was given before signed written consent. To ensure privacy, interviews took place in a private room at the college.

### 3.3. Analysis

The interviews were recorded by a digital audio recorder and transcribed verbatim for thematic analysis. According to Braun and Clarke’s approach to thematic analysis, accuracy of transcripts was checked before analysis [25]. The checking process was conducted by the same researcher based on familiarity with the data. All transcripts were then analyzed by using qualitative software, MAXQDA 2018. Initial sub-themes were generated through a sentence-by-sentence examination. Themes were searched and reviewed to fit with the data. The scope and content of each theme was defined and named concisely. The research team reviewed all themes and sub-themes to ensure cohesion with the interview data.

### 3.4. Trustworthiness

Before the process of data analysis, the senior qualitative researcher was invited to code the data. Three transcripts were randomly selected for the senior qualitative researcher and coded independently. The codes were then compared and discussed between the senior researcher and the research team to achieve mutual agreement on coding. A coding scheme was developed within the team to maintain consistency of analysis. In addition, the transcripts were returned to the participants to check for misinterpretation. Emerging themes and sub-themes were also returned for participants’ feedback. A draft copy was given to the senior researcher at the end of the study in order to detect any bias or inappropriate subjectivity.

### 3.5. Cohesion between Interviews

Cohesion between interviews and sub-themes was calculated by the similarity matrix in the qualitative software. Results showed that cohesion among the 21 interviews ranged from 0.14–1 with an average of 0.68. The low similarity coefficient (0.14) indicated that some sub-themes had not emerged across the interviews.

## 4. Results

The process of data collection stopped at the 21st interview since data saturation was suggested as no new information had emerged from the last three interviews. The interviews varied in time from 25–60 min. When the participants articulated their experience of clinical placement, 64 instances of challenge, such as poor nursing competency or relationship with preceptor, were reported among 21 participants. They believed challenges were common in the process of being a nurse. However, 10 participants reported strongly that these challenges influenced their social life resulting in a sense of dissatisfaction. Two themes, i.e., contributors to lack of social health, and manifestations of lack of social health, emerged, followed by several sub-themes (Table 2).

### 4.1. Contributors to Lack of Social Health

**Figure 1:**
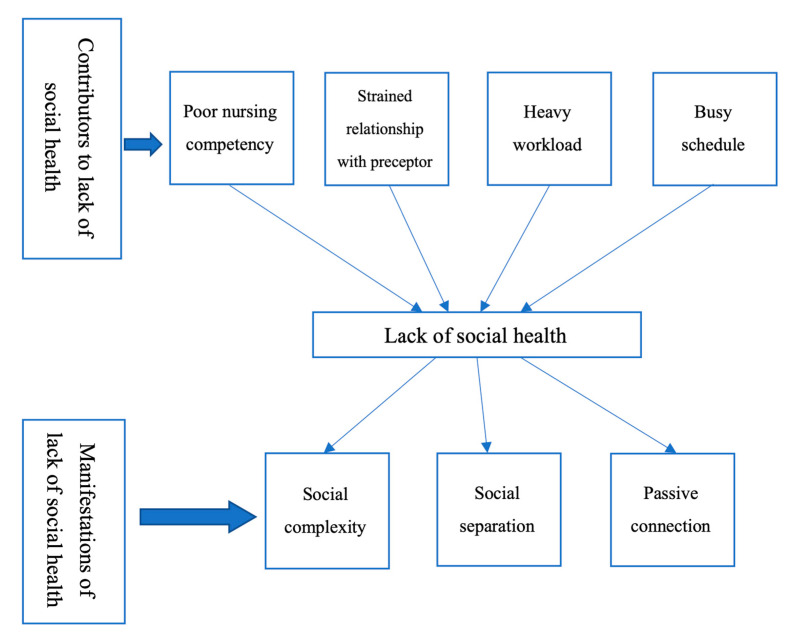
Contributors and manifestations of lack of social health.

Participants described their difficulties in clinical placement and four subthemes were identified (Table 2). Poor nursing competency was usually described among the participants. Some participants described that some preceptors did not accept suggestions about workflow, while some preceptors were unwilling to teach, since the students would leave the ward after several weeks. In addition, the work during placement and college resulted in participants experienced a tightened schedule. Although these challenges were common among the participants, some could create a balance in their daily life to overcome them at an early stage. However, some participants may require more time to overcome these challenges. Regarding interactions and social challenges in daily life, some participants developed an unhealthy sense of social status during the clinical placement as shown in Figure 1.

### 4.2. Manifestations of Lack of Social Health

When an individual experienced difficulty in the social challenges of daily life, an unhealthy social status would develop. Three sub-themes, i.e., social complexity, social separation, and passive connection, relating to the manifestations of lack of social health emerged as nursing students described their failure in meeting social challenges.

#### 4.2.1. Social Complexity

Participants agreed that they had to review their nursing knowledge during clinical placement, but they did not have sufficient time to do so. To some extent, they had to rearrange their daily life to put more time into preparation for the placement since it was the final clinical placement in their studies. The duration of clinical placement made them feel exhausted. In addition to their social life, their daily behaviors could become chaotic and out of control. Participants changed their routines with the aim of making their daily life more manageable. Some of them would deal with personal matters on their days off, but the day off was sometimes used to make further preparation for clinical placement.

#### 4.2.2. Social Separation

Some participants anticipated this unique characteristic of clinical placement. They excluded other activities as their schedule was filled by the roster of the clinical placement. The limited time space available in their schedule made them have to decide whether time was for themselves, family or friends. Moreover, participants might give up their own interests to focus on the placement.

Despite the foreseeable intensity of clinical placement, participants had thoughts about their future career in nursing. One participant described that a nurse had resigned as she wanted to have a baby. The frequent night shifts meant that she could not meet her husband to get pregnant, leading to a bad relationship with her husband and family. This participant portrayed her plan to work in the community as she highlighted the importance of family relationships and having a baby. Through reflection on the influence of the clinical placement on their social world, participants might make other choices after graduation.

#### 4.2.3. Passive Connection

Participants focused on the clinical placement and they did not actively connect with people around them. Fortunately, other people could actively create a supportive social atmosphere to help participants overcome challenges during the period of clinical placement. The stress evoked by clinical placement led to changes in participants. These changes might not be obvious, but their family and friends had a sense of salience which might have indirectly supported the participants during the clinical placement.

## 5. Discussion

Nursing students in Macau experience the same challenges during clinical placement as others around the world regardless of the uniqueness of their socioeconomic context [26]. However, the arrangement of a substantial proportion of the clinical placement in the final year raised a concern about their social health among nursing students. Through a learning process and role transformation, some students adapted well but others suffered decreased social health. The development of an unhealthy social status might be related to choice of study. Findings from a study of 400 students in healthcare major revealed that social health was significantly related to the degree of interest in pursuing healthcare as a major subject [27]. Nursing was not the first choice of study among 13 participants in this study, implying that their interest in nursing was not high, and these students might endure stress during placement resulting in a lack of social health.

A socially unhealthy status demonstrated a dissatisfaction with the social challenges faced by nursing students. The emerged sub-theme, social complexity, demonstrated a failure of social coherence [16]. Student’s social world became complicated and they lost a sense of what was happening around them. Their clinical placement was completely different from in previous years. Final-year students worked independently on a shift roster as a nurse, and switched to different specialties every 2-5 weeks to gain as much experience as possible. Students had to prepare in advance to fulfil the requirements of the next specialty when they were still having to address the issues in their current specialty. They needed to rearrange their routine to adapt to changes in both clinical placement and daily life. As a result, students had to minimize social contact with others to put more time into and concentrate on their clinical placement.

A further reduction of social contact made the students separate themselves from their social world. They lost the sense of belonging to society during clinical placement, resulting in dissatisfaction with social integration [16]. A moderate level of social health was noted among 288 staff nurses in a hospital in Iran [28]. As a dimension of social health, social integration was highlighted among the staff nurses at the lowest score. A low satisfaction with social integration represented a lack of belonging to the ward and/or the clinical environment [16]. A lack of belonging could made nurses quit the job or leave the career eventually [29]. In turn, nursing students would consider their future careers, influenced by their role model, the staff nurses [2].

Although some students could handle the challenges of clinical placement independently, the people around them also had a vital role in this adaption process. The sub-theme, passive connection, revealed a need from others to maintain their social health as staff nurses [17]. Social support, therefore, could help diminish the negative influence of the clinical environment on nursing students. In brief, the social health of nursing students should be at an optimal level to achieve the best learning outcomes during clinical placement [28].

On the other hand, this study revealed that nursing students seldomly sought help from others. A cross-sectional study of 343 Thai nursing students echoed that students were not actively seeking help from others [30]. Another study also found that young people did not seek help even if they held a positive attitude towards help-seeking behaviors [31]. Reluctance to seek help among the students in Macau had specific implications. Many students lived at home during clinical placement. Despite having support at hand, the students chose to ignore it, whereas students should be encouraged to approach family and friends for help, and family and friends should take time to look after student’s needs and offer help to those who choose to suffer silently.

### 5.1. Limitations

The first limitation of the study is that negative experience might be downplayed since the participants were told to achieve certain requirements during clinical placement by their preceptors. Secondly, the study accessed students from one nursing college in Macau. Most clinical placements were conducted in one hospital and students might have different experience if their placements were conducted in other hospitals. Some students who were unwilling to participate in this study might also experience difficulties that they did not want to tell others about. Although the interviewer had no conflict with the student’s academic or clinical results, students might worry that negative comments might lead to a negative impression from the college. This might be a reason that only ten students described their sense of dissatisfaction with social life during the clinical placement.

### 5.2. Implications

Several implications were suggested for nursing education and clinical practice. For nursing education, observation of nurse’s work could be organized for first-year nursing students and secondary school students to increase their interest in the nursing profession. Observation could help indecisive secondary school students to choose the nursing profession and increase the confidence of first-year nursing students to stay in the nursing profession. This strategy might also be applicable to other forms of healthcare education to increase students’ interest in the profession. On the other hand, a week of preparation could be arranged during the clinical placement to provide a space for students to revise their knowledge and skills for the coming short two weeks specialty. In addition, the challenges encountered in clinical placement aligned with a previous systematic review [26]. The nursing college could arrange a regular briefing, maybe bi-weekly, as a supportive strategy to explore students’ concerns about the clinical placement. The college could then communicate with the clinical preceptors about the students’ concerns and work together to facilitate student’s learning and lower the negative influence of clinical placement on student’s daily lives. For instance, the college and the preceptors in each ward could develop a list of basic requirements for students to follow. This might help students prepare in a focused manner for each ward.

A high intensity of work was commonly described in the study and this could lead to burnout eventually [29,32]. Mindfulness-based intervention was suggested as effective in promoting mental health and decreasing the effect of burnout [33,34]. A session of mindfulness intervention could be arranged on each shift to diminish the effect of stress on nurses and nursing students. A regular mindfulness workshop could also be arranged for healthcare providers, including the students on the ward, to diminish their stress and promote mental well-being. Although the effect of mindfulness interventions on social health was not examined, it could diminish the impact of social unhealth. In addition, a change in the clinical environment might diminish the risk of burnout [35]. For instance, a review showed that soothing music could reduce stress that when employed as background music on the ward [36]. This might help nurses and nursing students develop a sense of belonging to the ward with a result of improved social health. Further study is suggested to examine the effect of different strategies on social health.

## 6. Conclusions

This study contributes to knowledge of social health and its effect on QOL among nursing students during clinical placement. The findings show that the common challenges faced in clinical placement, such as poor nursing competency and strained relationships with preceptor, contribute negatively to the student’s social health in terms of social complexity, social separation and passive connection, resulting possibly in leaving the profession after graduation. Several strategies are suggested to potentially improve social health, such as increasing interest in the profession and the rearrangement of clinical placement. Although the study targeted final-year nursing students, decreased social health during clinical placement may appear in junior nursing students, which is rarely explored in previous studies. Further study is needed to explore whether students in different years and/or in different nursing programs experience the same influence on social health during clinical placement. The examination of suggested strategies as well as the development of proactive strategies to improve social health during clinical placement needs further investigation.

## Figures and Tables

**Table 1:** Participant characteristics.

Characteristics	N (%) or Range
Gender Female	21 (100%)
Age Range	22–25
Race Chinese	21 (100%)
Application to study nursing Self-apply Referred by secondary school	17 (81%) 4 (19%)
Nursing as the first choice Yes No	8 (38%) 13 (62%)

**Table 2:** Emerged themes from the interviews.

Themes	Sub-Themes	Exemplary Quotes
Contributors to lack of social health	Poor nursing competency	…I am afraid of being questioned [about the knowledge] by my preceptor. (Participant 21)
		…I was not used to work independently as I worked in group over the last three years. (Participant 02)
	Strained relationship with preceptor	…If you had different viewpoints from your preceptor, conflict could arise. You needed to know how to communicate with your preceptor. (Participant 18)
		…The preceptor did not spend much time to know you. She followed what she was used to do in clinical environment. You needed to follow her steps. (Participant 05)
	Heavy workload	…The stressors came from everywhere such as patient’s condition, workload in ward and projects from college. (Participant 06)
	Busy schedule	…I found I had less leisure time since I was not used to the arrangement of placement and life. (Participant 10)
Manifestations of lack of social health	Social complexity	…At the beginning (of clinical placement), it was very stressful. I put most of my effort on studying, preparation for placement and sleep. I could not do other things. (Participant 07) …I needed to make preparation… Because of the roster, I did not have a day off for months. (Participant 03)
	Social separation	…I told my friends not to find me in this year. I had clinical placements and work in shift…I would call you once I was free and they kept silent…My friends were at the bottom, while the (clinical placement related) workgroups were on the top of the list in the communication application. (Participant 14) …I joined a dancing group when I was in secondary school…I kept active in the group…I had thought of withdrawal because of the clinical placement in this year. (Participant 8)
	Passive connection	…Although they (family members) did not participate in my learning process, they would support me in another way. For instance, they made some delicious foods and created a silent environment. They used different strategies to facilitate my learning. (Participant 8) …My family celebrated with me, but I did not know it was the Easter day that day. (Participant 3)

## Supplementary Materials

The following are available online at https://www.mdpi.com/1660-4601/17/18/6921/s1, Interview guide.
