# Supplementary material for: “My Friends are at the Bottom of My Schedule”: A Qualitative Study on Social Health among Nursing Students during Clinical Placement
Source: Int J Environ Res Public Health. 2020 Sep 22;17(18):6921. doi: 10.3390/ijerph17186921 (PMC7559577; doi:10.3390/ijerph17186921)
Supplement: Supplementary file 1 [file ijerph-17-06921-s001.pdf]

Interview guide

1. In review of the final-year clinical placement, what have you learned? What is the difference for you when comparing the placement this year to those in the previous three years?

2. How would you prepare yourself for the final-year clinical placement? Is there any difference in preparation to the previous clinical placements?

3. Did you face any difficulty related to the clinical placement this year? Or what impressed you the most in the final-year clinical placement? (e.g. a thank you card from a patient)

4. For the previous three years, your clinical placements were guided by the teachers from the college. You are now guided by the clinical preceptors in each ward. What do you think about the preceptors? Any good or bad aspects in terms of the clinical teaching methods with either of the teachers from the college or the clinical preceptors?

5. Now that all the placement has been finished and you are having a holiday, what is your plan for your future career and why would you make this plan?
